# Supplementary figures and images for: Breeding and hibernation of captive meadow jumping mice (Zapus hudsonius)
Source: PLoS One. 2021 May 10;16(5):e0240706. doi: 10.1371/journal.pone.0240706 (PMC8109813; doi:10.1371/journal.pone.0240706)

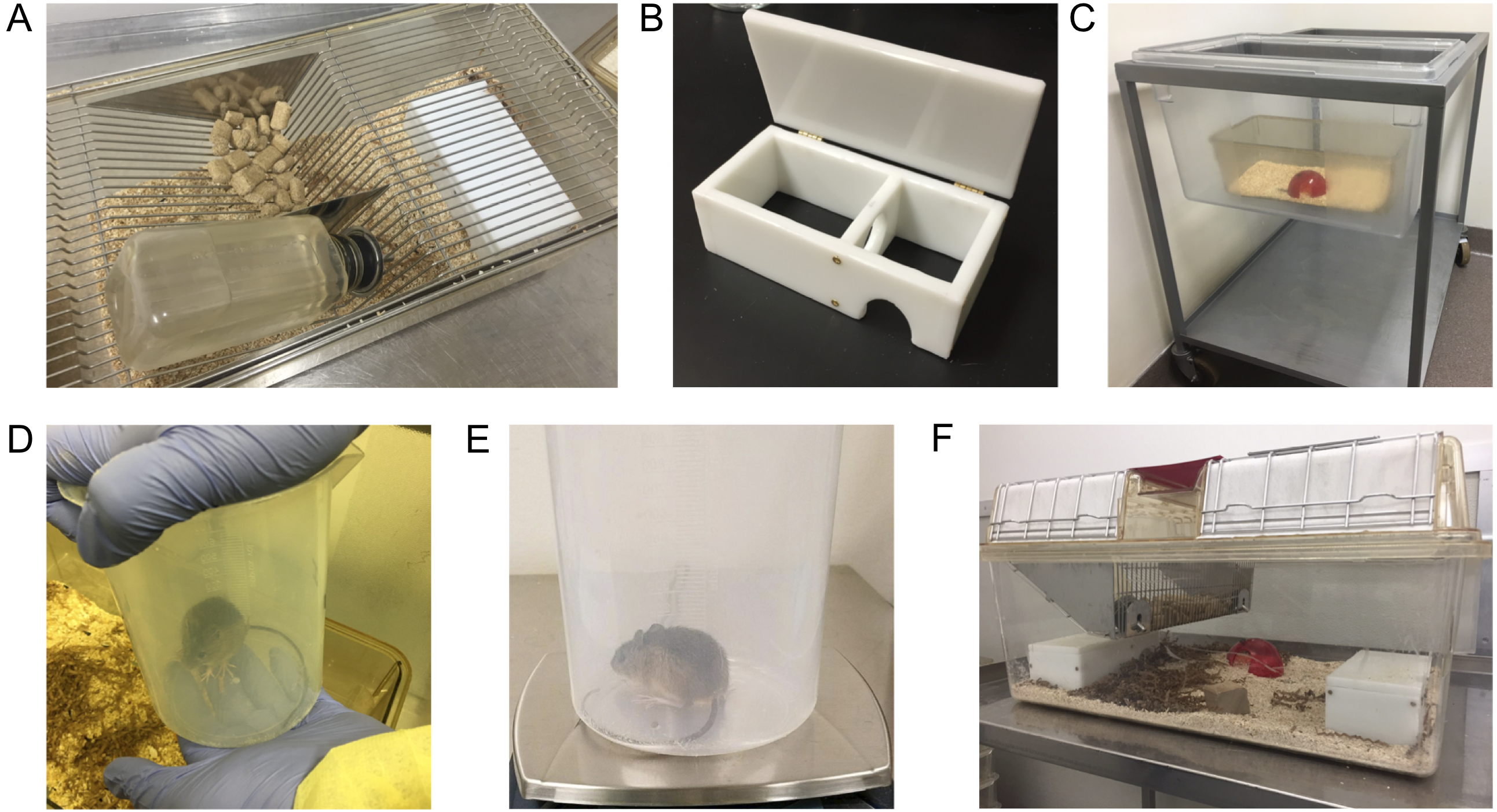

Supplement: S1 Fig — (A) A cage used for single housing of Zapus; the white nest box fits inside this cage for hibernation experiments. (Cage lid not shown). (B) Interior of nest box, showing entrance, hinged lid, and two interior chambers. (C) Placing the cage inside a large plastic bin provides containment during animal handling. This demonstration cage contains the type of red plastic shelter provided during routine housing. (D) We use a plastic beaker for low-stress animal handling when restraint is not required. (E) Body mass can be conveniently determined with the animal contained inside a plastic beaker. (F) The large cages used for breeding contain two nest boxes and additional enrichment. (TIFF) [file pone.0240706.s001.tiff]

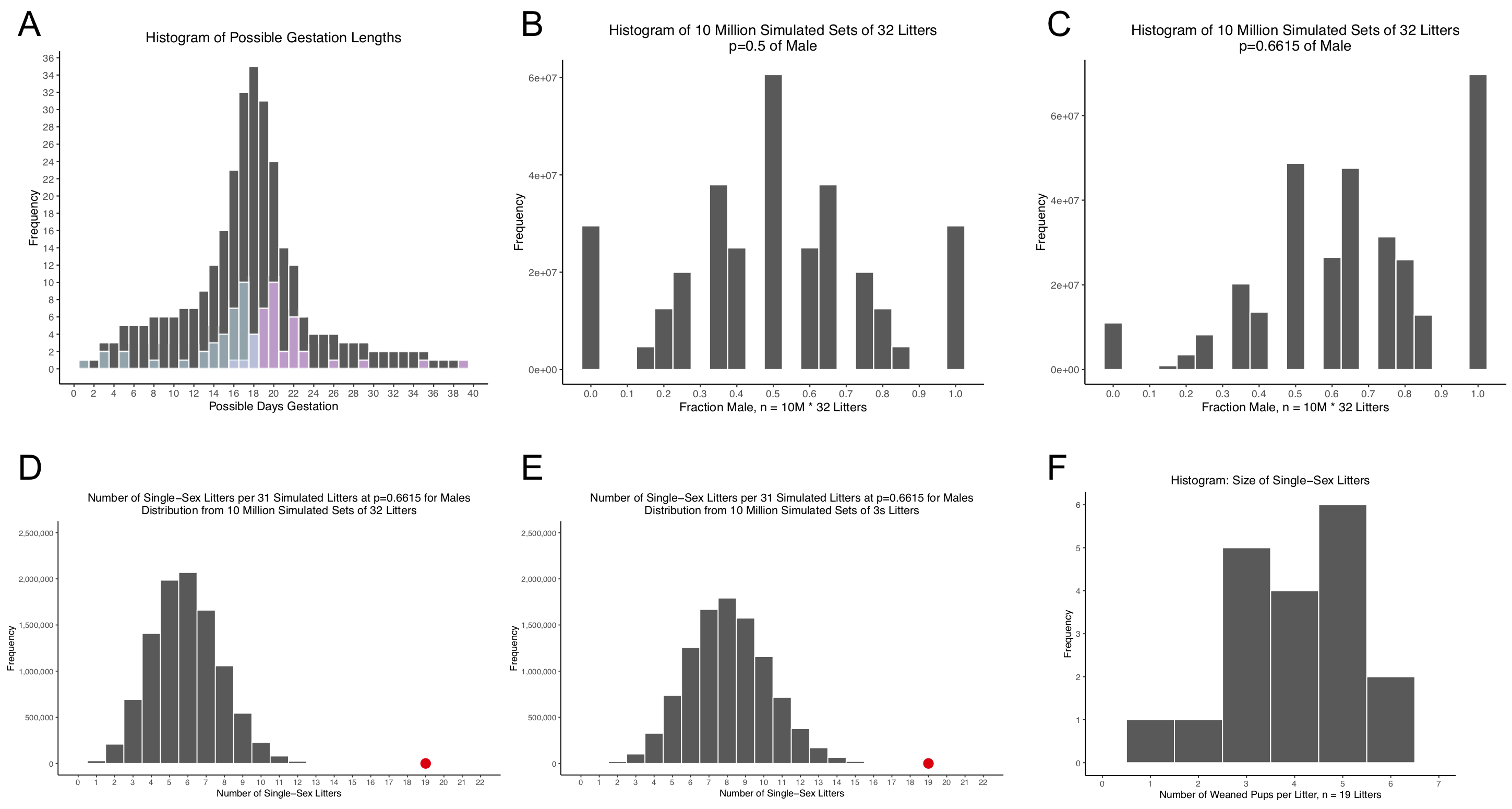

Supplement: S2 Fig — (A) Histogram of all possible gestation lengths as determined from the pairing and separation dates of 39 successful pairs of meadow jumping mice. The lower and upper bounds (shortest and longest possible gestation lengths) are overlaid in blue and pink, respectively. (B) Histogram showing number of litters by litter sex ratio, from 10 million sets of 32 litters that were simulated assuming an underlying sex ratio (fraction male) of 0.5. (C) Histogram showing number of litters by litter sex ratio, from 10 million sets of 32 litters that were simulated assuming an underlying sex ratio (fraction male) of 0.6615385, the ratio observed in our colony. (D) Plot showing the distribution of the number of single-sex litters obtained from 10 million sets of 32 litters simulated assuming an underlying sex ratio (fraction male) of 0.5. A red circle is placed at 19 single-sex litters, the number observed in our colony. (E) Plot showing the distribution of the number of single-sex litters obtained from 10 million sets of 32 litters simulated assuming an underlying sex ratio (fraction male) of 0.6615385, the ratio observed in our colony. A red circle is placed at 19 single-sex litters, the number observed in our colony. (F) A histogram of the sizes of 19 single-sex litters that occurred in our colony, by number of weaned pups per litter. The mean is 4 pups per litter. (TIFF) [file pone.0240706.s002.tiff]

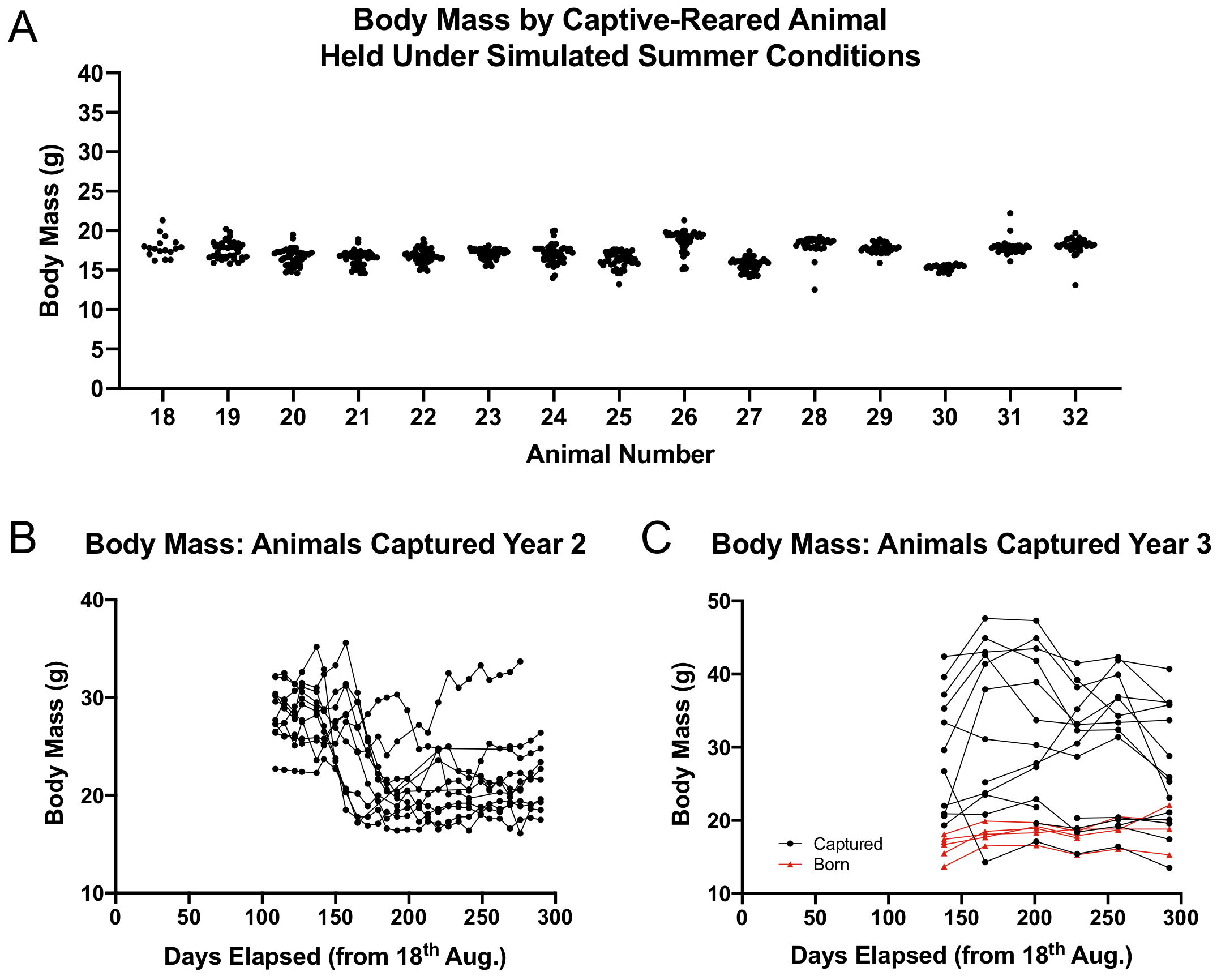

Supplement: S3 Fig — (A) Each body mass measurement for each captive-reared animal from the time series shown in Fig 3A. Zapus born in captivity and held under simulated summer conditions (20° C, 16L:8D) maintained a relatively constant body mass for over one year. (B) Body mass over time for the animals captured during the second year of trapping and held at 20° C and 16L:8D photoperiod. For logistical reasons, data collection started some time after capture. These animals had fattened post-capture and then almost all of them spontaneously returned to a lean summer condition. (C) Body mass over time for the animals captured during the third year of trapping (black lines), or born to a captured female (red lines), and held at 16L:8D. For logistical reasons, data collection started some time after capture. Captured animals had fattened while captive-born animals maintained summer weight. (TIFF) [file pone.0240706.s003.tiff]

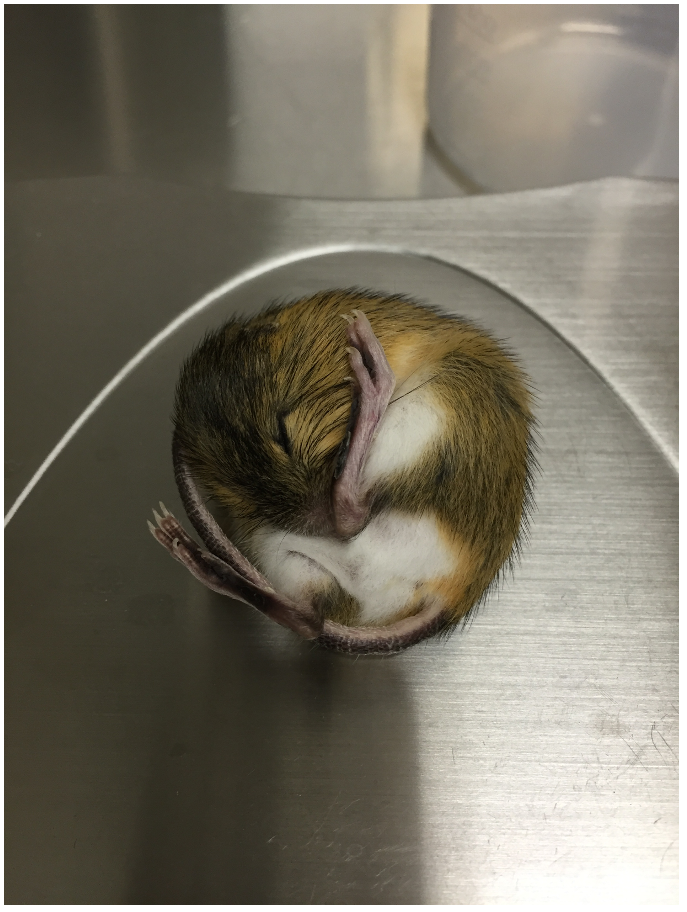

Supplement: S4 Fig — This animal has rolled slightly onto its side on the laboratory scale; the head and feet are directly underneath the animal when torpid in the hibernaculum. If handling must occur, accidental arousals can be minimized through rapid and gentle handling that does not uncurl the animal or turn it upside down. (TIF) [file pone.0240706.s004.tif]

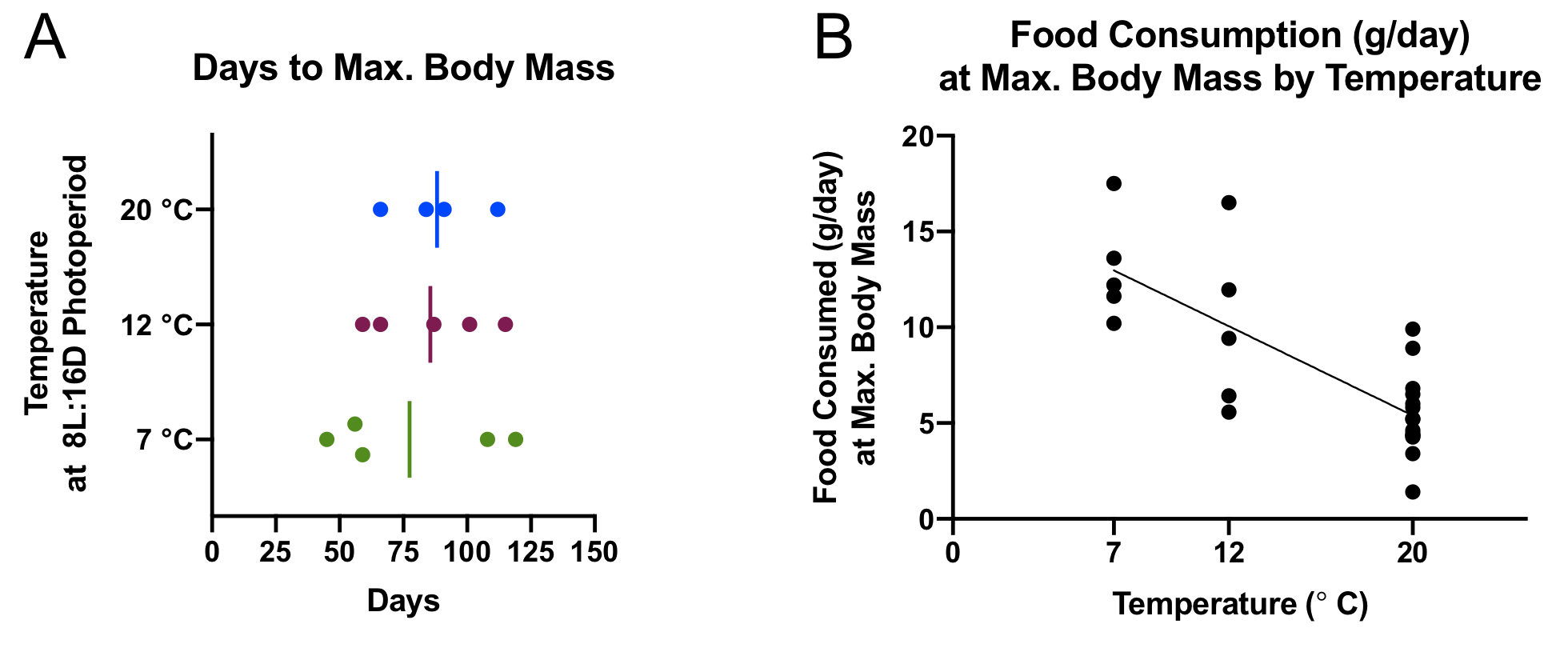

Supplement: S5 Fig — (A) Days from start of hibernation induction to maximum attained body mass for each induction temperature. (B) Relationship between temperature and food consumed per day at maximum body mass during hibernation induction. Control animals are included at 20° C. Food consumption is increased at lower temperatures, as indicated by the negative slope of the best fit line. (TIFF) [file pone.0240706.s005.tiff]

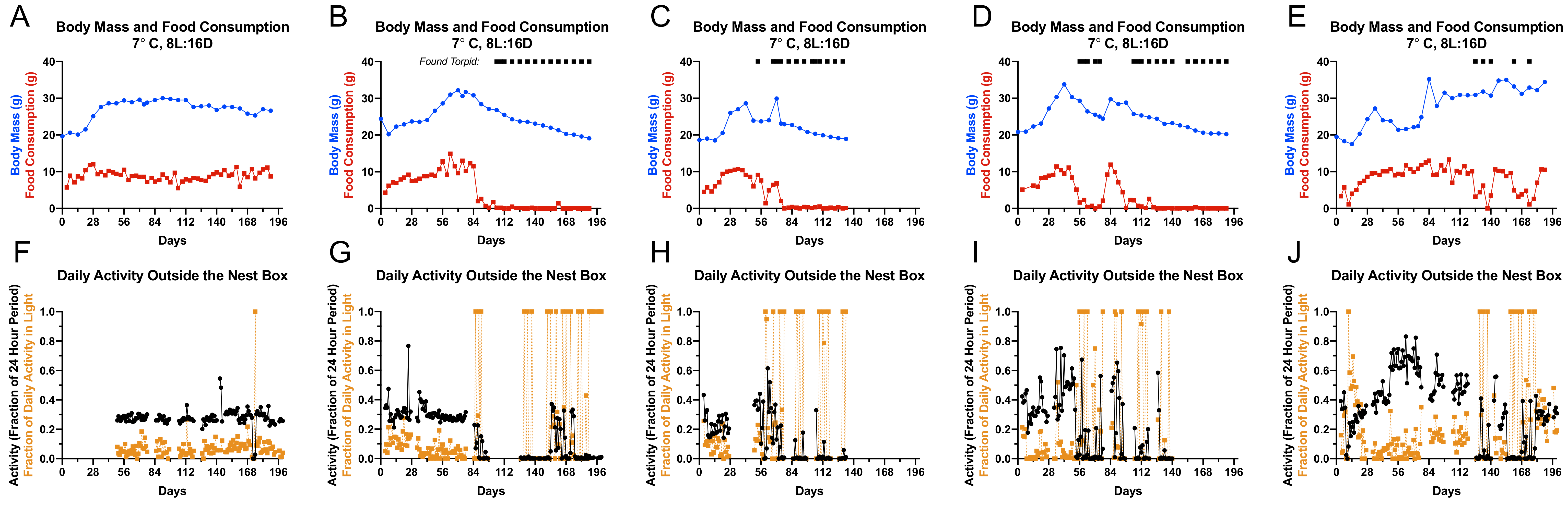

Supplement: S6 Fig — (A-E) Body mass and daily food consumption are shown for animals induced to hibernate in simulated winter conditions (7° C and 8L:16D). When applicable, black squares indicate the days the animal was found torpid. (F-J) Daily activity outside of the nest box as recorded by passive infrared motion detector. Activity is represented as the fraction of each 24 hour day spent active by the animal (black lines and symbols), and the fraction of total activity occurring in the light phase is shown by orange lines and symbols. Before the hibernation interval, the animals typically spend less than half of the day active, with only a minor portion of total activity occurring during the light phase. Following the onset of hibernation, a larger proportion of the reduced level of activity occurs during the light phase, consistent with disruption of the circadian rhythm. (TIFF) [file pone.0240706.s006.tiff]
