# Supplementary material for: Breeding and hibernation of captive meadow jumping mice (Zapus hudsonius)
Source: PLoS One. 2021 May 10;16(5):e0240706. doi: 10.1371/journal.pone.0240706 (PMC8109813; doi:10.1371/journal.pone.0240706)
Supplement: S2 Table — (DOCX) [file pone.0240706.s008.docx]

**S2 Table. Number of animals fattening and found torpid by experimental condition.**

| **Condition** | **Fattening** | **Found Torpid** | **Total Animals** |
| --- | --- | --- | --- |
| Control (20º C, 16L/8D) | 0 | 0 | 10 |
| Induction (20º C, 8L/16D) | 4 | 2 | 5 |
| Induction (12º C, 8L/16D) | 5 | 4 | 5 |
| Induction (7º C, 8L/16D) | 5 | 4 | 5 |
